# Cell type-specific potential pathogenic genes and functional pathways in Alzheimer’s Disease

**DOI:** 10.1186/s12883-021-02407-1

**Published:** 2021-10-02

**Authors:** Xiao-Lan Wang, Lianjian Li

**Affiliations:** 1 Department of Nephrology, Union Hospital, Tongji Medical College, Huazhong University of Science and Technology, Wuhan, China; 2 Université de Strasbourg, Strasbourg, France; 3 Department of Surgery, Hubei Provincial Hospital of Traditional Chinese Medicine, Wuhan, 430061 China; 4 Hubei Province Academy of Traditional Chinese Medicine, Wuhan, 430076 China

**Keywords:** Alzheimer's disease, cell type-specific, transcriptomic, mitochondrial dysfunction, estrogen signaling pathway

## Abstract

**Background:**

Alzheimer's disease (AD) is a pervasive age-related and highly heritable neurodegenerative disorder but has no effective therapy. The complex cellular microenvironment in the AD brain impedes our understanding of pathogenesis. Thus, a comprehensive investigation of cell type-specific responses in AD is crucial to provide precise molecular and cellular targets for therapeutic development.

**Methods:**

Here, we integrated analyzed 4,441 differentially expressed genes (DEGs) that were identified from 263,370 single-cells in cortex samples by single-nucleus RNA sequencing (snRNA-seq) between 42 AD-pathology subjects and 39 normal controls within 3 studies. DEGs were analyzed in microglia, astrocytes, oligodendrocytes, excitatory neurons, inhibitory neurons, and endothelial cells, respectively. In each cell type, we identified both common DEGs which were observed in all 3 studies, and overlapping DEGs which have been seen in at least 2 studies. Firstly, we showed the common DEGs expression and explained the biological functions by comparing with existing literature or multil-omics signaling pathways knowledgebase. We then determined the significant modules and hub genes, and explored the biological processes using the overlapping DEGs. Finally, we identified the common and distinct dysregulated pathways using overall DEGs and overlapping DEGs in a cell type-specific manner.

**Results:**

Up-regulated LINGO1 has been seen in both oligodendrocytes and excitatory neurons across 3 studies. Interestingly, genes enriched in the mitochondrial module were up-regulated across all cell types, which indicates mitochondrial dysfunction in the AD brain. The estrogen signaling pathway seems to be the most common pathway that is disrupted in AD.

**Conclusion:**

Together, these analyses provide detailed information of cell type-specific and overall transcriptional changes and pathways underlying the human AD-pathology. These findings may provide important insights for drug development to tackle this disease.

**Supplementary Information:**

The online version contains supplementary material available at 10.1186/s12883-021-02407-1.

## Background

Alzheimer’s disease (AD) is the most common form of dementia characterized by the accumulation of extracellular amyloid-β (Aβ) and neurofibrillary tangles, and progressive synaptic and neuronal dysfunction and degeneration [1–3]. However, AD pathogenesis is not restricted to neurons and increasing evidence suggests that multiple cell type interactions in the brain promote AD development [4–8]. Immune response-induced chronic neuroinflammation has been considered as a critical component in the progression of AD, but anti-inflammatory drug candidates still failed in clinical trials [9–11]. The complex interplay of cells in the brain may limit our understanding of the pathological mechanisms of AD. Thus, it’s important to clearly understand how different cell types contribute to AD progression and outcome.

Microglia serve as the brain’s resident macrophages with immune-modulating and phagocytic capability. Compelling evidence revealed an extended microglial gene network in AD [12–14] and has firmly linked microglia to Aβ deposition and synaptic loss [15, 16]. Microglia in AD, a double-edged sword, protect against the initiation of AD, and their activation-induced inflammation also leads to AD progression [8, 17, 18]. Astrocytes provide trophic and metabolic support to neurons in the brain, however, microglia secreted inflammatory cytokines activate the neurotoxic A1 astrocytes, which cooperate with microglia to mediate complement-dependent neuronal loss [6, 19]. Activated astrocytes have been found from both postmortem AD patients and animal models [4, 20]. Moreover, a recent in vivo study showed that astrocytes are also involved in the engulfment of apoptotic neurons to maintain brain homeostasis [21], which may play an important role in the AD brain.

Genome-wide associated studies have indicated that most of the risk factors for AD are expressed by microglia (such as APOE and TREM2) and astrocytes (such as CLU), which are associated with immune response, as well as oligodendrocytes (such as BIN1) [13, 20, 22]. Oligodendrocytes produce the myelin that ensheaths axons, provide trophic and metabolic support to neurons, and regulate neuronal connectivity. Reduced oligodendrocytes and myelin have been observed consistently in AD, which may be caused by Aβ toxicity and increased inflammation and oxidative stress in the brain [5, 23, 24]. Moreover, in most AD patients, Aβ deposition has been seen around perivascular, which leads to endothelial cell dysfunction and death, increases the permeability of the blood-brain barrier (BBB) and neuroinflammation, and further contributes to AD progression [7, 25, 26].

Therefore, it’s critical to comprehensively analyze the transcriptomic changes in different cell types in the AD brain. Here, we integrated analyzed the differentially expressed genes (DEGs) identified from cortex samples of AD individuals and normal controls in 3 single-nucleus RNA sequencing (snRNA-seq) studies (Mathys et al, Grubman et al, and Lau et al) [27–29]. Our comprehensive molecular profiling of the human AD brain may figure out the cell type-specific and overall pathogenic genes and disease-associated signaling pathways, which may provide therapeutic targets for AD.

## Methods

### Data Source and identification of overlapping DEGs

Transcriptome results were from age and sex-matched AD-pathology individuals and normal controls. In the study of Mathys et al, 24 prefrontal cortex samples were used in each group and statistical enrichment for sets of marker genes [30, 31] was used for the identification of cell types. 1,031 DEGs were identified from 70,634 single-cells transcriptomes in 6 cell types (FDR-corrected *P <* 0.01 in two-sided Wilcoxon-rank-sum test, absolute log fold change> 0.25, and FDR-corrected P < 0.05 in Poisson mixed model). Gene expression results of Mathys et al were obtained from Supplementary Material 4. To be noticed, mitochondrial genes have been removed in the study of Mathys et al. In the study of Lau et al, 21 prefrontal cortex samples (AD = 12, normal controls = 9) were included for transcriptome analysis, and Seurat was used to identify the cell types. 2,190 DEGs were identified from 169,496 nuclei in 6 cell types (adjusted *P* < 0.1, absolute log2 fold change ≥ 0.1). DEGs results were obtained from Supplementary file 4. In the study of Grubman et al, BRETIGEA [32] was used to identify the cell types. Gene expression results were downloaded from http://adsn.ddnetbio.com, and FDR < 0.01, absolute log fold change > 0.5 were considered as statistically significant differences between AD and normal controls (n = 6 entorhinal cortex samples per group). 1,726 DEGs were defined from 13,214 nuclei in 8 classified cell types. DEGs that were used in this study have been added in the Supplemental Files.

Venn Diagram (http://bioinformatics.psb.ugent.be/webtools/Venn/) was used to calculate and draw a venn map for each cell type, and overlapping DEGs were retained for further analysis. The log2 fold change of overlapping DEGs was used to generate Heatmap using R software (version 3.4.210).

### Construction of PPI network and module analysis

Overlapping DEGs was used to construct protein-protein interaction (PPI) network [33, 34] using STRING analysis (http://string-db.org Version:11.0) and was further analyzed in Cytoscape software (3.7.2 version) by both cytoHubba and Molecular Complex Detection (MCODE) plugins in Cytoscape to select significant hub genes and modules from the PPI network [35, 36] in each cell type. Hub genes were ranked by MCC method in cytoHubba and presented by nodes with different colors in figures (red to yellow means ranking from high essential to essential). Edges mean interactions, reactions, or regulations among nodes in the network. Modules were identified by MCODE and presented with a circle layout. The selection criteria of MCODE: degree cutoff = 2, node score cutoff = 0.2, and k-score = 2, max. Depth = 100.

### Signaling pathways analysis

The Signaling Pathway Project (https://www.signalingpathways.org/index.jsf), a multil-omics knowledgebase for cellular signaling pathway [37, 38], was used to evaluate the cellular signaling pathways that single differentially expressed gene involved in. Human transcriptomics datasets were chosen, and FDR significant cut-off = 1E-02.

### GO biological process and KEGG pathways enrichment analysis

The annotation function of GO biological process of overlapping DEGs was carried out using the online DAVID Bioinformatics database 6.8 [39], which is a database resource for understanding high-level functions and utilities of the genes. KEGG pathway enrichment analysis of all DEGs and overlapping DEGs [40] was performed in the online DAVID Bioinformatics database 6.8. *P*-value < 0.05 was considered as significant differences for both GO analysis and KEGG pathway enrichment analysis. *P*-value, fold enrichment, and gene counts in each term were used to create a Bubble chart in R software (version 3.4.210).

## Results

### Disrupted immune response, energy supply, and oxidative stress, as well as reduced protein degradation in microglia

**Fig. 1:**
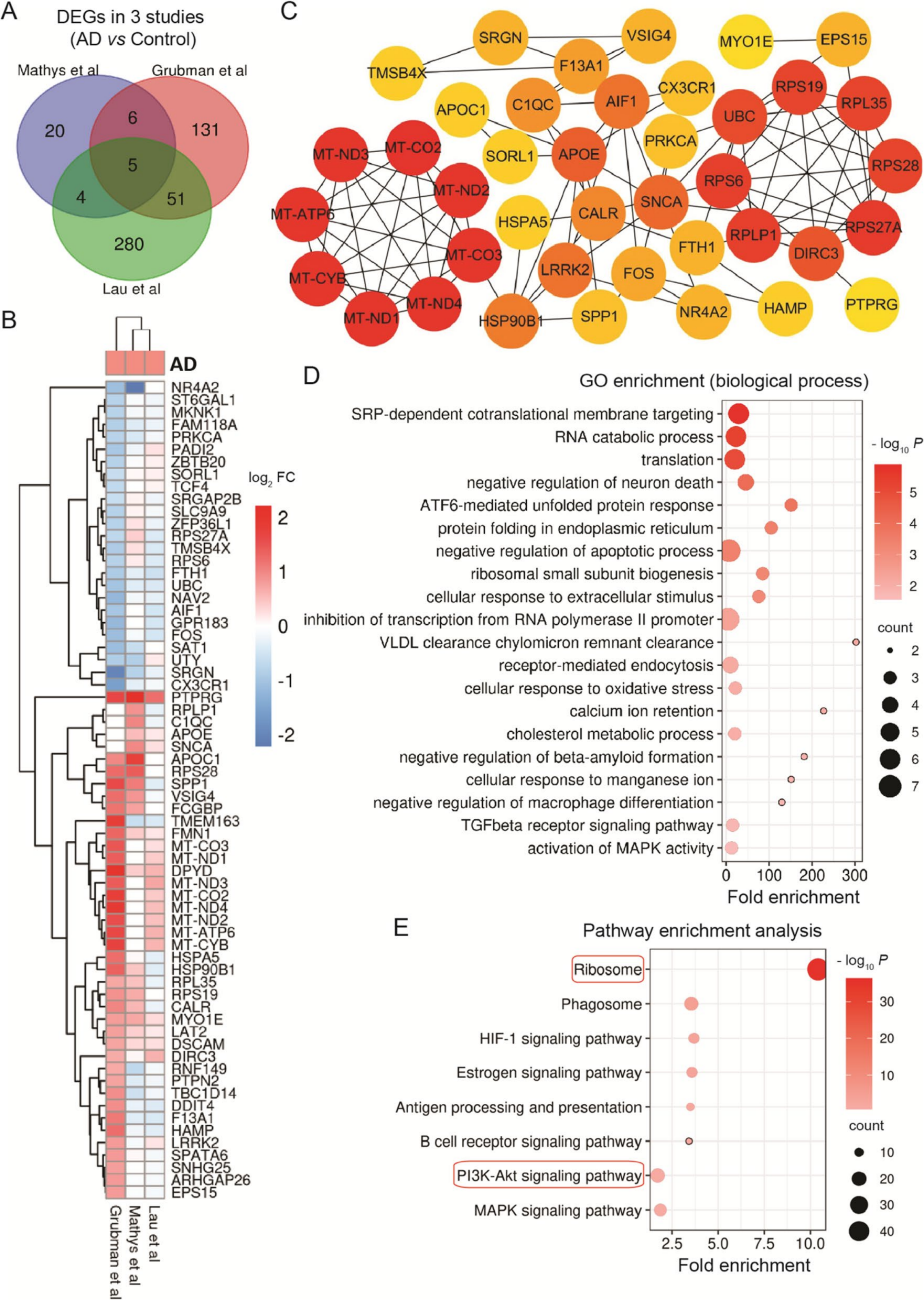
Microglia. Overlapping differentially expressed genes (DEGs) and pathways in microglia from 3 studies. **A** Venn diagram showing overlapping DEGs detected in microglia. **B** List of overlapping DEGs at least in 2 studies. Heatmap colored by gene expression in each study (red: up-regulated, blue: down-regulated). **C** PPI network of overlapping DEGs. Significant modules are indicated in circle layout. Significance of hub genes is indicated by color; red to yellow means significance from high to low. **D** Top 20 significant gene ontology (GO) terms for overlapping DEGs. **E** Significant KEGG pathways of DEGs in microglia. Red circles show pathways generated using overlapping DEGs. In bubble charts, significance is indicated by color, and the gene number of DEGs is indicated by the size of a dot

Human genetic studies pointed out a key role of microglia in the pathogenesis of AD [13]. To identify the potential pathogenic genes and cellular processes in microglia, we integrated analyzed the DEGs in 3 studies. We observed 5 common DEGs, and only 2 genes showed the concordant change in microglial cells (Fig.1A and B, and Table 1). Concordant up-regulated PTPRG and MYO1E, and discordant SPP1, VSIG4, and RNF149 are involved in inflammation [41–45]. The further investigation of these common genes in multil-omics signaling pathways knowledgebase showed that except RNF149, the other genes are also involved in the estrogen signaling pathway in human tissue [46, 47]. These findings indicate that the dysregulated immune response and the involvement of the estrogen signaling pathway may be the common characteristic of microglia in AD.

**Table 1:**
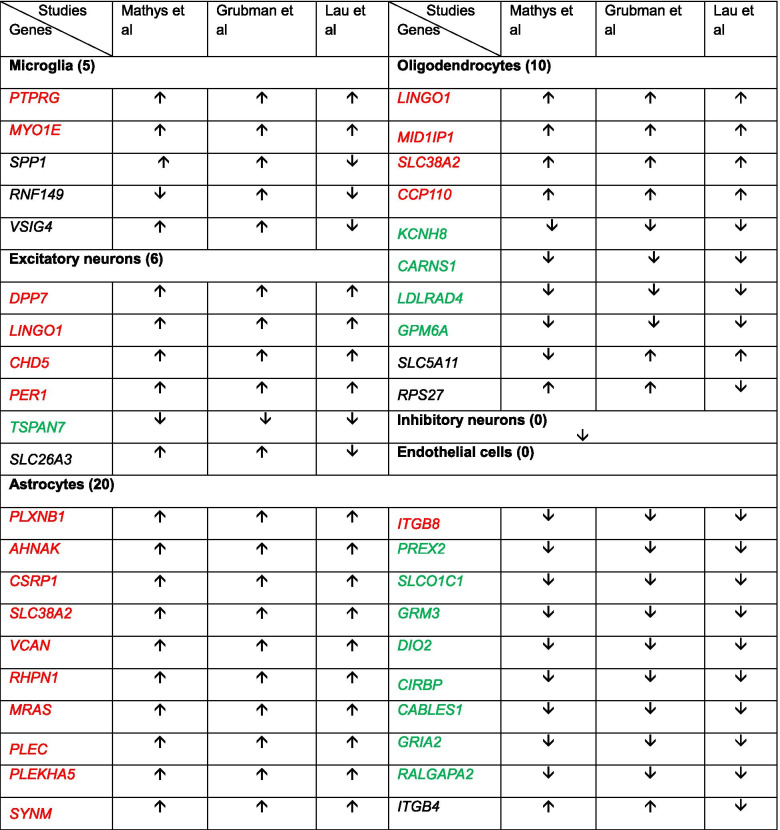
Common DEGs in 3 studies (AD versus Control)

Red color means concordant up-regulated genes and green color means concordant down-regulated genes in 3 studies

There were another 61 overlapping DEGs among 3 studies, including up-regulated APOE and APOC1 (Fig.1A and B), the risk factors for developing AD [48, 49], suggesting the importance of these DEGs in AD-pathology progression. We then identified the important modules and hub genes among the total 66 overlapping DEGs. The top-ranked genes (up-regulated MT-ND1–4, MT-CO2, MT-CO3, MT-ATP6, and MT-CYB) in the PPI network were enriched in the mitochondrial module (8 nodes and 28 edges) from both Grubman et al and Lau et al studies (Fig.1B and C). The changed sugar metabolism and mitochondrial function have also been observed in a large-scale proteomic analysis of AD brain, especially in glial cells [50]. GO enrichment analysis showed that the genes-RPS19, RPS28, RPL35, RPS27A, RPLP1, and RPS6 that involved in the second module (8 nodes and 27 edges) were enriched in SRP-dependent cotranslational protein targeting to membrane, mRNA catabolic process, translational initiation, and ribosomal processes (Fig.1B-D). While hub genes-UBC and RPS27A, which involved in protein ubiquitination to eliminate the toxic protein aggregation, including ribosomes, were down-regulated in microglia (Fig.1B and C) [51–54]. Moreover, UBC and RPS27A were also involved in negative regulation of apoptotic and transcription processes, TGF beta signaling pathway, inflammatory signaling pathways, and activation of MAPK activity in AD (Fig.1D). DIRC3, a lncRNA, was up-regulated, which may interact with peroxisome proliferator-activated receptors (PPARs) according to the multil-omics signaling pathways knowledgebase (Fig.1B and C).

Microglial cells in AD were also enriched for genes involved in the regulation of neuron death, lipid-related clearance, response to oxidative stress (such as up-regulated LRRK2, APOE, and SNCA), response to extracellular stimulus, microglia activation (such as down-regulated AIF1), unfolded protein response, protein folding in endoplasmic reticulum (ER), endocytosis, and calcium ion retention (such as HSP90B1 and CALR) (Fig.1B-D). The KEGG pathway enrichment analysis of the DEGs showed that ribosome, phagosome, antigen processing and presentation, HIF-1 signaling pathway, estrogen signaling pathway, B cell receptor signaling pathway, PI3K-Akt signaling pathway, and MAPK signaling pathway were enriched in microglial cells (Fig.1E). Especially the ribosome and PI3K-Akt signaling pathway were enriched in at least 2 studies (Fig.1E).

### Disturbed cellular homeostasis in astrocytes and deficit of neuronal support

**Fig. 2:**
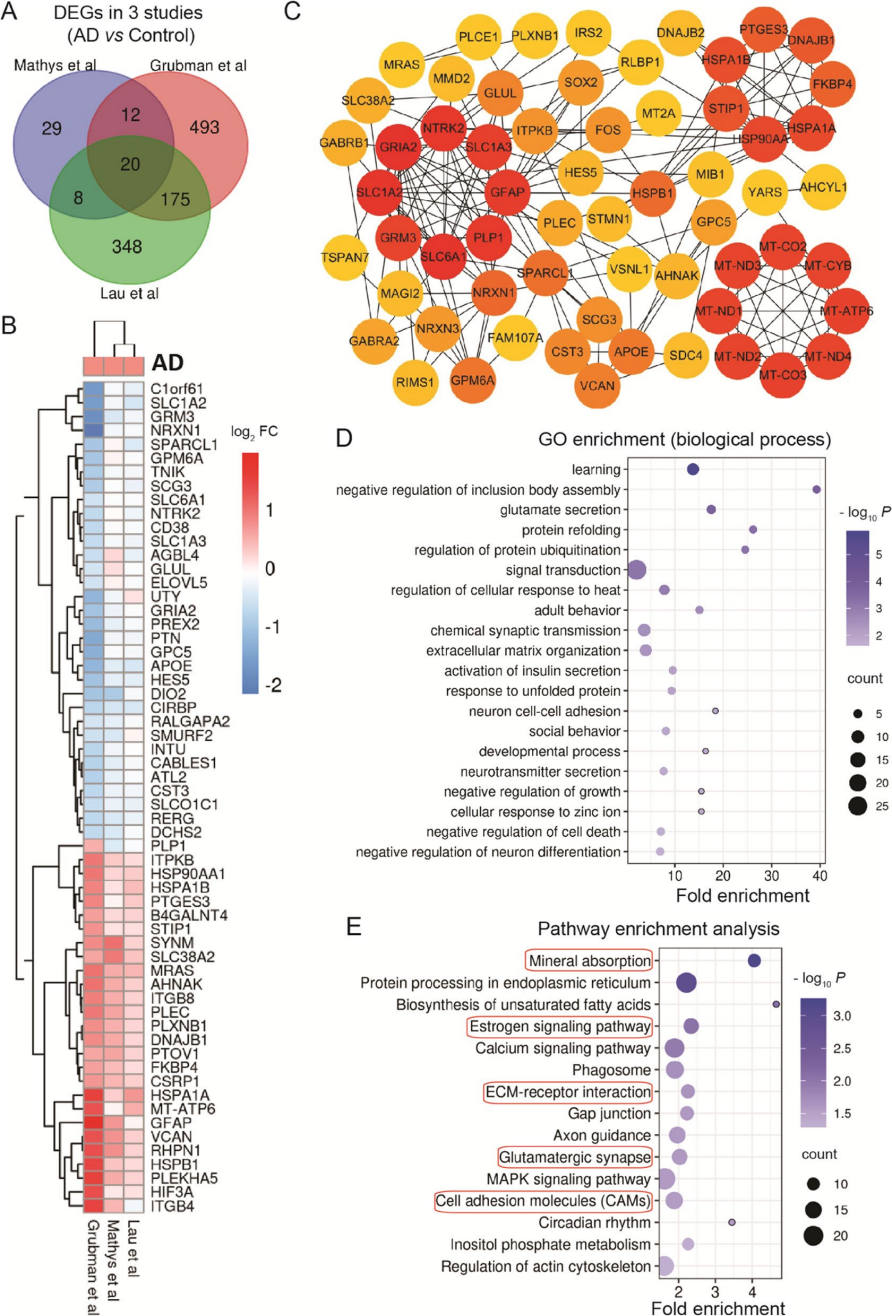
Astrocytes. Overlapping DEGs and pathways in astrocytes. **A** Venn diagram showing overlapping DEGs. **B** List of partial overlapping DEGs at least in 2 studies. **C** PPI network of partial overlapping DEGs. **D** Top 20 significant gene ontology (GO) terms for overlapping DEGs. **E** Significant KEGG pathways of DEGs. Red circles show pathways generated using overlapping DEGs

Astrocytes support neuronal functions, including recycling of neural transmitters, modulation of synaptic transmission, stimulation of synaptogenesis, regulation of ion concentration in the extracellular space, and maintenance of BBB. Here, we observed 20 common DEGs in 3 studies, and 19 genes (11 up-regulated and 8 down-regulated) showed concerted changes in astrocytes (Fig.2A and B, and Table 1). These genes were enriched for glutamate receptors (down-regulated GRIA2 and GRM3), glutamate secretion (up-regulated SLC38A2), thyroid hormone perturbation (down-regulated SLCO1C1 and DIO2) [55, 56], extracellular matrix organization (ITGB8, ITGB4, and VCAN), circadian clock regulation (down-regulated CIRBP) [57], permeability of BBB (up-regulated PLEKHA5) [58], and cell proliferation (up-regulated RHPN1) [59], indicating the dysfunction of astrocytes and neurons in AD. While we also observed the altered PLXNB1, MRAS, CSRP1, AHNAK, SYNM, PREX2, RALGAPA2, CABLES1, and PLEC expression, which has been described in previous human AD studies (Fig.2B) [60–67].

Overlapping DEGs among 3 studies showed high concerted gene expression in astrocytes (Fig.2B). In overlapping DEGs constructed PPI network, the most significantly changed genes were enriched in mitochondrial module, as we observed in microglia. GO enrichment showed that the hub genes (SLC6A1, SLC1A2, SLC1A3, NTRK2, GRIA2, GRM3, PLP1, SPARCL1, GPM6A, and NRXN1), which were mostly involved in the second module, were enriched for processes related to learning, glutamate secretion, signal transduction, chemical synaptic transmission, neuron cell-cell adhesion, neurotransmitter secretion, synaptic assembly (Fig.2C and D). These alterations indicate the disrupted neuronal signal transduction in AD. In the third module, genes-DNAJB1, HSP90AA1, HSPA1B, PTGES3, FKBP4, HSPA1A, HSPB1, and CST3, were enriched for negative regulation of inclusion body assembly, cellular response to heat, regulation of protein ubiquitination, protein refolding, unfolded protein, and negative regulation of cell death (Fig.2C and D). In addition, some hub genes were enriched in extracellular matrix organization (VCAN and GFAP), activation of insulin secretion and glutamate catabolic process (GLUL), and negative regulation of neuron differentiation (SOX2) (Fig.2C and D). To be noticed that APOE which involved in retinoid metabolic process, triglyceride homeostasis, and NMDA glutamate receptor clustering was down-regulated in astrocytes (Fig.2B and D).

Further KEGG pathway enrichment showed that the overlapping DEGs were mainly enriched for pathways, such as mineral absorption, cell adhesion molecules, glutamatergic synapse, GABAergic synapse, extracellular matrix (ECM)-receptor interaction, and estrogen signaling pathway (Fig.2E). Moreover, overall DEGs in astrocytes were also involved in protein processing in ER, biosynthesis of unsaturated fatty acids, calcium signaling pathway, phagosome, gap junction, axon guidance, MAPK signaling pathway, circadian rhythm, inositol phosphate metabolism, and regulation of actin cytoskeleton (Fig.2E).

### Increased oxidative stress in oligodendrocytes

**Fig. 3:**
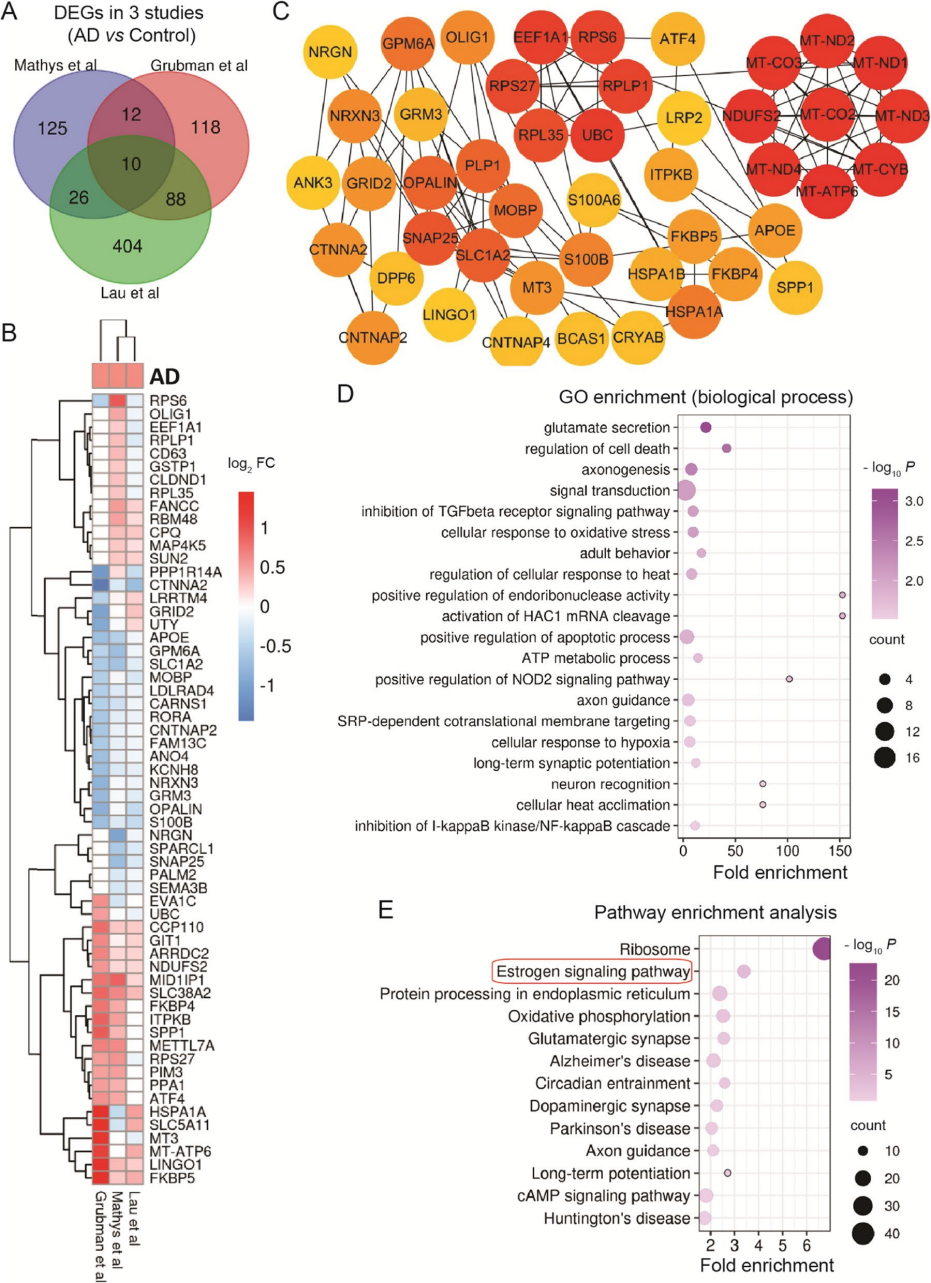
Oligodendrocytes. Overlapping DEGs and pathways in oligodendrocytes. **A** Venn diagram showing DEGs. **B** List of partial overlapping DEGs at least in 2 studies. **C** PPI network of partial overlapping DEGs. **D** Top 20 significant gene ontology (GO) terms for overlapping DEGs. **E** Significant KEGG pathways of DEGs. Red circles show pathways generated using overlapping DEGs

Oligodendrocytes dysfunction has been associated with neurodegenerative disease and neuroimaging studies showed that the myelin loss happened in the preclinical phase of AD [68, 69]. Here, we observed 10 common DEGs in 3 studies (Fig.3A). Up-regulated LINGO1 was involved in axonogenesis and signal transduction, while MID1IP1 and SLC38A2 were related to AD pathology, which has been reported in previous studies [70, 71]. Multil-omics signaling pathways knowledgebase showed that CCP110, KCNH8, CARNS1, LDLRAD4, and GPM6A might be involved in the cell cycle, immune response, and estrogen receptor pathway [42].

In overlapping DEGs constructed PPI network, the top-ranked module (9 nodes and 36 edges) was enriched for up-regulated mitochondria related-genes (MT-ND1–4, MT-CO2, MT-CO3, MT-ATP6, MT-CYB, and NDUFS2) (Fig.3B and C). Genes in the second module (dysregulated RPS27, RPS35, RPS6, UBC, and RPLP1) and together with the hub genes (down-regulated S100B and CTNNA2) were enriched in SRP-dependent cotranslational protein targeting to membrane, mRNA catabolic process, and regulation of apoptotic process (Fig.3B-D). Down-regulated S100B, CTNNA2, SNAP25, SLC1A2, CNTNAP2, and NRXN3 were involved in axonogenesis, glutamate secretion, long-term synaptic potentiation, and adult behavior (Fig.3B-D). HSPA1A and MT3 were involved in cellular response to oxidative stress and hypoxia. Besides, down-regulated DEGs were also enriched in glutamate receptor (GRID2), stabilizing the myelin sheath (MOBP), and promoting oligodendrocyte terminal differentiation (OPALIN) (Fig.3B-D). The up-regulated PLP1 and ITPKB have been considered as risk factors of AD in previous studies [72, 73]. These altered hub genes and related biological processes indicate the increased cellular oxidative stress and injury of oligodendrocytes, which further affect neural transmission.

Using the DEGs, we explored the KEGG pathway enrichment in oligodendrocytes, including ribosome, estrogen signaling pathway, protein processing in ER, oxidative phosphorylation, glutamatergic and dopaminergic synapse, circadian entrainment, axon guidance, long-term potentiation, cAMP signaling pathway, AD, Parkinson's disease, and Huntington's disease (Fig.3E). It seems that these DEGs include the transcriptional changes of other neurodegenerative diseases.

### Dysfunction of cellular metabolism and protein degradation in excitatory neurons

**Fig. 4:**
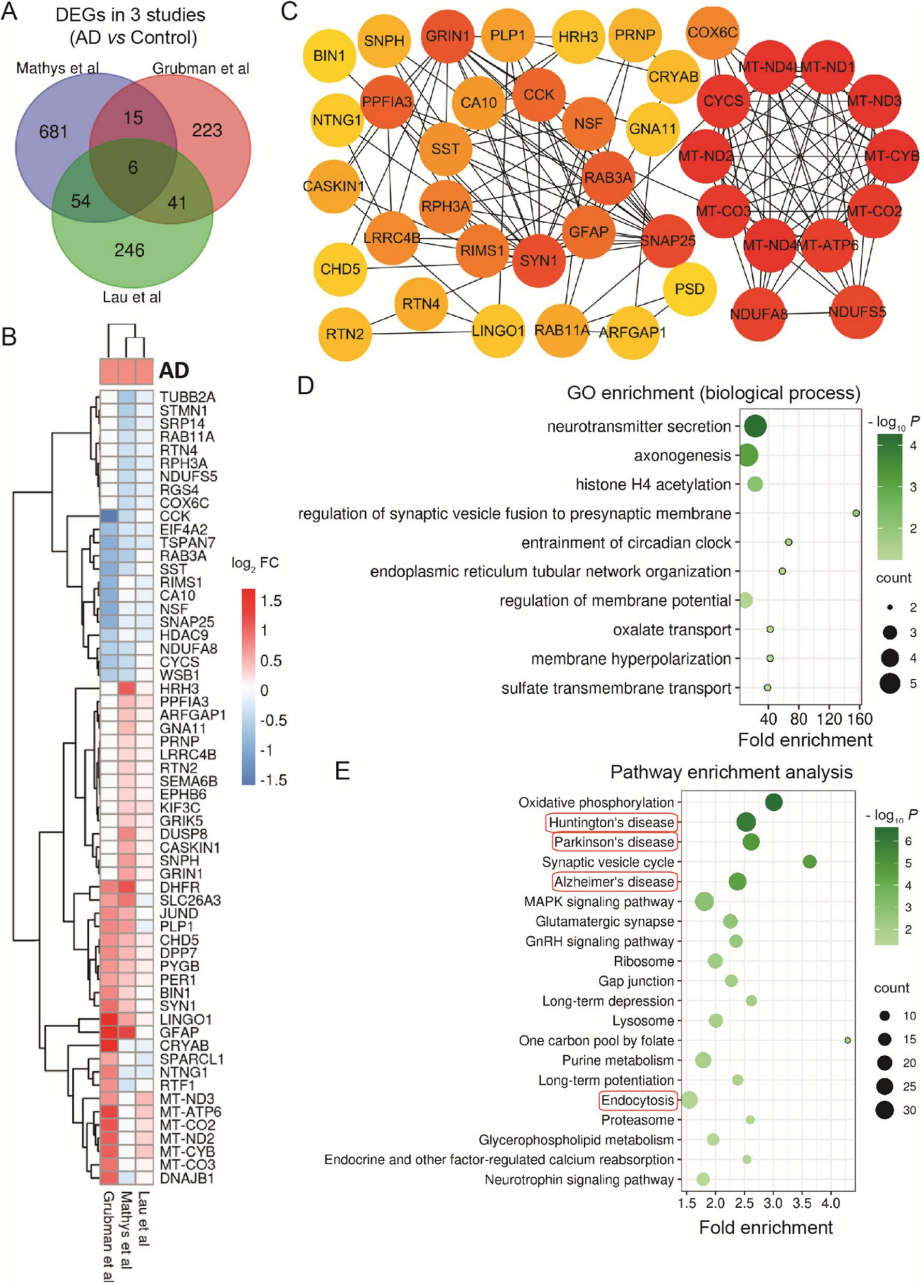
Neurons (Excitatory). Overlapping DEGs and pathways in excitatory neurons. **A** Venn diagram showing DEGs. **B** List of partial overlapping DEGs at least in 2 studies. **C** PPI network of partial overlapping DEGs. **D** Significant gene ontology (GO) terms for overlapping DEGs. **E** Significant KEGG pathways of DEGs. Red circles show pathways generated using overlapping DEGs

Synaptic dysfunction and neuronal loss are the main characteristics of AD. In excitatory neurons, we observed 6 common DEGs in 3 studies (Fig.4A). These overlapping DEGs were enriched in axonogenesis (up-regulated LINGO1), histone H4 acetylation (up-regulated CHD5 and PER1) [74], and entrainment of circadian clock (PER1) (Fig.4B-D). TSPAN7, regulation of spine maturation and AMPA receptor trafficking, was down-regulated in excitatory neurons [75]. While SLC26A3 was enriched in regulation of membrane potential, membrane hyperpolarization, oxalate transport, and sulfate transmembrane transport. Multil-omics signaling pathways knowledgebase showed that the up-regulated DPP7 was involved in innate immune response (Fig.4B-D).

Moreover, in the PPI network of overlapping DEGs, the top-ranked genes (up-regulated MT-ND1–4, MT-ND4L, MT-CO2, MT-CO3, MT-ATP6, MT-CYB, and CYCS) were enriched in mitochondrial module (with 10 nodes and 45 edges) (Fig.4B-C). While the genes-NDUFA8, NDUFS5, and COX6C that related to mitochondrial electron transport chain were down-regulated (Fig.4B-C). These changes in mitochondria indicate the disorder of oxidative phosphorylation. Among the hub gene, down-regulated SNAP25 has been considered as a potential biomarker in AD [76]. Up-regulated GRIN1, a glutamate receptor, was enriched in the regulation of membrane potential. Up-regulated PPFIA3 together with the genes (SYN1 and RAB3A) from the second module were enriched in neurotransmitter secretion. Down-regulated RAB3A and CCK were enriched in axonogenesis and regulation of synaptic vesicle fusion to presynaptic membrane. RIMS1, associated with synaptic transmission, and LRRC4B, related to cell adhesion, were disturbed (Fig.4B-D). Multil-omics signaling pathways knowledgebase showed that down-regulated NSF was related to inflammation. Up-regulated SYN1, down-regulated RAB3A, RPH3A, SST, and CCK were closely associated with AD development [77–81] (Fig.4B-C). Together, these changed gene expressions indicate the deficit of synaptic function in excitatory neurons.

The KEGG pathway enrichment analysis showed that the overlapping DEGs were enriched in AD, Parkinson's disease, Huntington's disease, and endocytosis (Fig.4E), suggesting these DEGs may be the common gene set involved in neurodegenerative diseases. Overall DEGs were also involved in oxidative phosphorylation, synaptic vesicle cycle, MAPK signaling pathway, glutamatergic synapse, GnRH signaling pathway, ribosome, gap junction, long-term depression, lysosome, one carbon pool by folate, purine metabolism, long-term potentiation, proteasome, glycerophospholipid metabolism, endocrine and other factor-regulated calcium reabsorption, and neurotrophin signaling pathway (Fig.4E).

### Mitochondrial dysfunction in inhibitory neurons

**Fig. 5:**
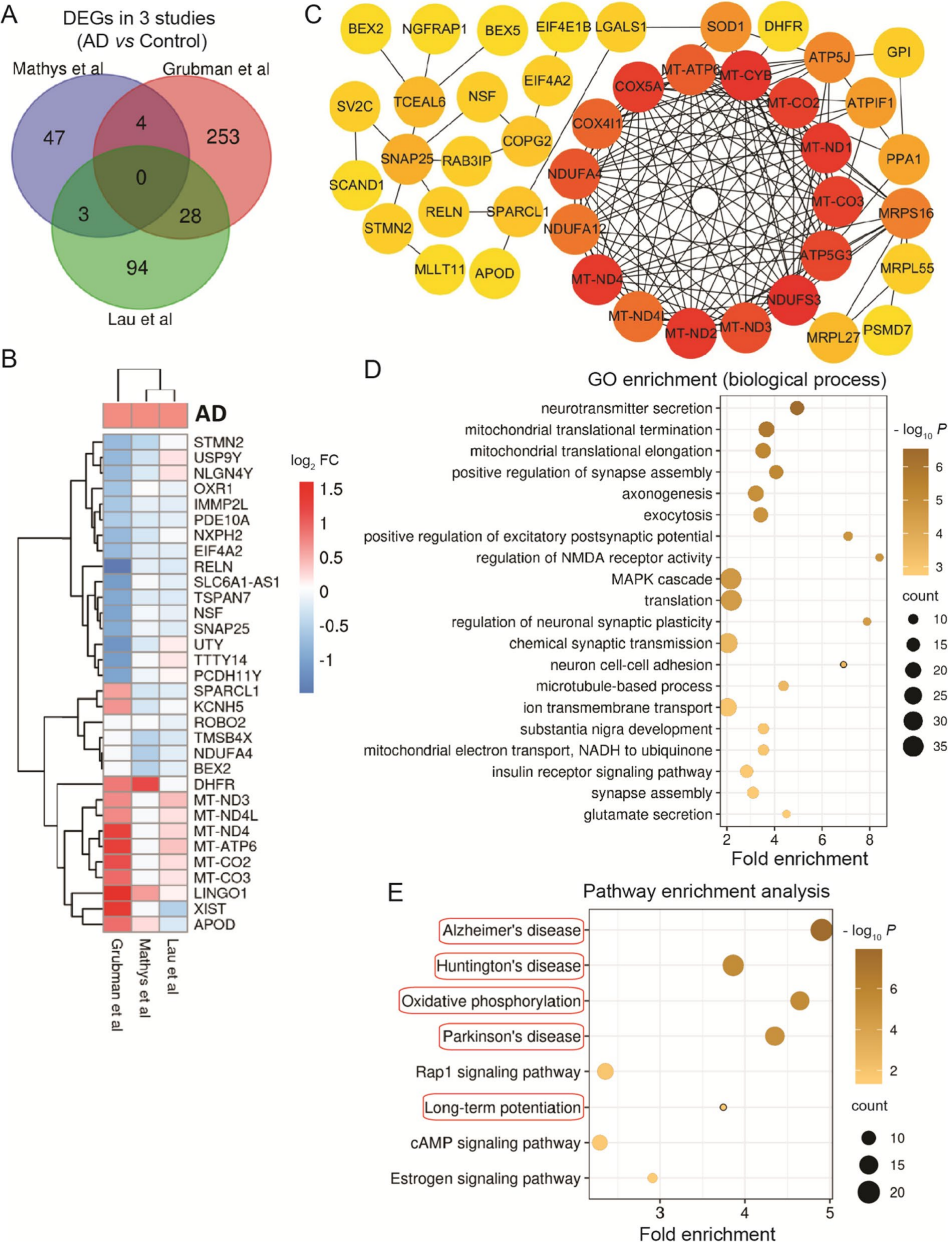
Neurons (Inhibitory). Overlapping DEGs and pathways in inhibitory neurons. **A** Venn diagram showing DEGs. **B** List of overlapping DEGs between any 2 studies. **C** PPI network of overlapping DEGs. **D** Top 20 significant gene ontology (GO) terms for overlapping DEGs. **E** Significant KEGG pathways of DEGs. Red circles show pathways generated using overlapping DEGs

In inhibitory neurons, there were no common DEGs in 3 studies (Fig.5A). The 35 overlapping DEGs among 3 studies were used to construct the PPI network. The significant module included up-regulated mitochondrial genes and down-regulated mitochondrial respiratory chain-related genes (NDUFA4, NDUFA12, NDUFS3, and COX4I1) (Fig.5B and C). These DEGs together with down-regulated ATPIF1, ATP5J, and MRPS16 indicate the mitochondrial dysfunction in inhibitory neurons. SOD1, which is involved in response to reactive oxygen species, was down-regulated (Fig.5B-D). In addition, the overlapping DEGs were also enriched in neurotransmitter secretion, axonogenesis (up-regulated LINGO1), exocytosis, NMDA receptor activity, MAPK cascade, and cell adhesion (Fig.5B-D). Overlapping DEGs were enriched in pathways including AD, Huntington's disease, Parkinson's disease, and long-term potentiation. Overall DEGs were also involved in Rap1 signaling pathway, cAMP signaling pathway, and estrogen signaling pathway (Fig.5E).

### Increased energy metabolism and immune response in endothelial cells

**Fig. 6:**
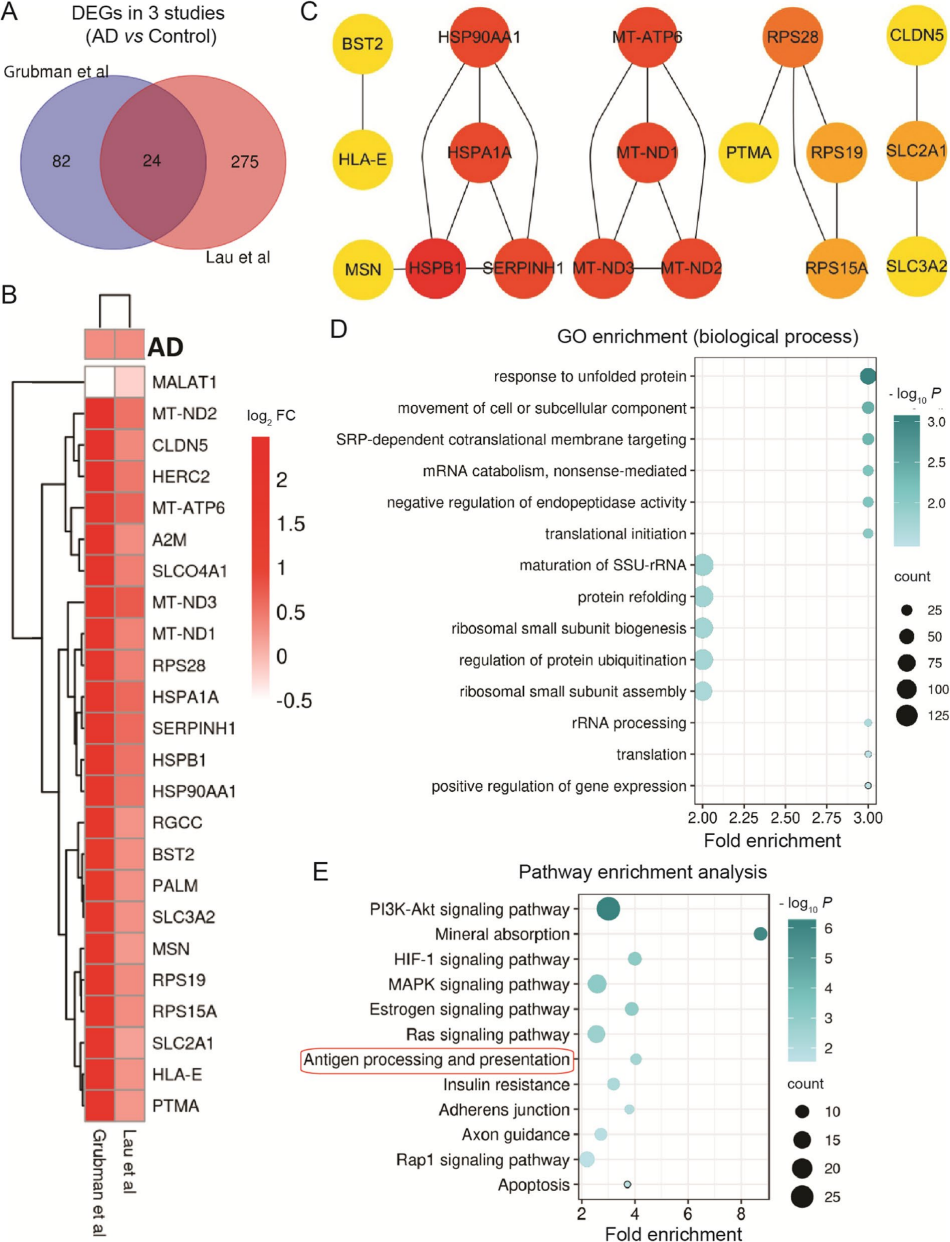
Endothelial cells. Overlapping DEGs and pathways in endothelial cells. **A** Venn diagram showing DEGs. **B** List of overlapping DEGs between 2 studies. **C** PPI network of overlapping DEGs. **D** Significant gene ontology (GO) terms for overlapping DEGs. **E** Significant KEGG pathways of DEGs. Red circles show pathways generated using overlapping DEGs

Endothelial cells are a central element to form the BBB and regulate molecular transport into the brain. BBB impairments have been seen in the preclinical stages of the AD brains [82]. Here, only 2 studies classified the endothelial cells and we have 24 overlapping DEGs (Fig.6A). Except for MALAT1, the overlapping DEGs showed high concordant up-regulation in endothelial cells (Fig.6B). Interestingly, mitochondria and nutrient transporter related genes, such as glucose transporter-SLC2A1, intracellular calcium and amino acid transporter-SLC3A2, transporter activity regulator-SLCO4A1, and the physical barrier-CLDN5, were significantly increased, suggesting the increased uptake of nutrients and energy production in AD brain [83–85] (Fig.6B).

GO analysis showed that DEGs were also enriched in response to unfolded protein (HSP90AA1, SERPINH1, and HSPB1), movement of cell or subcellular component (HSPB1, MSN, and PALM), SRP-dependent cotranslational protein targeting to membrane, mRNA catabolism, nonsense-mediated, translational initiation, rRNA processing and translation (RPS28, RPS15A, and RPS19), maturation of SSU-rRNA, ribosomal small subunit processing (RPS28 and RPS19), protein refolding, regulation of protein ubiquitination (HSP90AA1 and HSPA1A), negative regulation of endopeptidase activity (BST2, SERPINH1, and A2M), and positive regulation of gene expression (RGCC, MSN, and HSPA1A) (Fig.6C and D). Also, the overlapping DEGs were enriched in immune response (PTMA, HLA-E, and MALAT1) and ubiquitin-protein ligase binding (HERC2) [86, 87]. These changes indicate the cellular stress and protein degradation in endothelial cells.

The common KEGG pathway between the 2 studies was antigen processing and presentation (Fig.6E). The overall DEGs were also involved in PI3K-Akt signaling pathway, mineral absorption, HIF-1 signaling pathway, MAPK signaling pathway, estrogen signaling pathway, ras signaling pathway, insulin resistance, adherens junction, axon guidance, rap1 signaling pathway, and apoptosis (Fig.6E).

## Discussion

In recent decades, it has been reported that numerous risk factors, different cell types, and complex signaling pathways are involved in AD pathogenesis. However, there is currently no effective therapy to cure the disease. Here, we integrated analyzed the cell type-specific transcriptomic and functional changes to understand the common and distinct molecule changes and networks across different cell types in AD.

### Common molecule and pathways changes in AD

Up-regulated LINGO1, a negative regulator of neuronal processes, was the only differential gene observed in multiple cell types across all studies, which has been predicted to be a potential target of AD therapy [29, 88]. Moreover, we found the coordinated up-regulation of mitochondrial genes across all cell types in the studies of Lau et al and Grubman et al. Increasing evidence indicates the mitochondrial disorder in AD brain [7, 89–91]. Mitochondria, as the center of cellular metabolism, not only provide enough energy supply to maintain the essential cellular processes but may cause mitochondrial-related oxidative damage. Mitochondrial dysfunction could impair astrocyte's neuroprotective effect and synaptic activity [92]. Besides, altered energy metabolism also implicates mitochondrial dysfunction, which has been seen in microglia, astrocytes, and oligodendrocytes [5, 50]. Metabolic processes affect the immune response and oxidative stress and vice versa [93–96]; the unbalance of this complex interplay aggravates the oxidative stress, dysfunction of cellular metabolism, and immune response as we have observed in AD brain, especially in microglia, oligodendrocytes, and endothelial cells. Overall, the mitochondrial transcriptome alterations are the most significant and consistent changes that cross all cell types, while the mitochondrial dysfunction has also been approved in a recent proteomic study in the human AD brain [90].

The overall transcriptomic changes in each cell type revealed more possible impaired pathways involved in AD-pathology. Common KEGG pathways enriched in different cell types were shown in Table 2. The dysregulated MAPK signaling pathway has been observed in microglia, astrocytes, excitatory neurons, and endothelial cells. Inhibition of the p38 MAPK signaling pathway to treat AD has been suggested since decades ago [97, 98]. The antigen processing and presentation, HIF-1 signaling pathway, and PI3K-Akt signaling pathway were enriched in microglia and Endothelial cells. These pathways together with mitochondrial and estrogen signaling pathways form a complex network and play an extensive and important role in AD pathology [99]. Phagosome was enriched in microglia and astrocytes, but the phagocytosis of astrocytes could be compensation for microglial dysfunction [100]. The estrogen signaling pathway seems to be the most common pathway that is disrupted in AD.

**Table 2:** Common KEGG pathways in different cell types of AD brain

Pathways	Cell types
Estrogen signaling pathway	Mic, Astro, Oligo, Neuron (In), Endo
MAPK signaling pathway	Mic, Astro, Neuron (Ex), Endo
Ribosome	Mic, Oligo, Neuron (Ex)
Glutamatergic synapse	Astro, Oligo, Neuron (Ex)
Axon guidance	Astro, Oligo, Endo
Oxidative phosphorylation	Oligo, Neuron (Ex), Neuron (In)
Long-term potentiation	Oligo, Neuron (Ex), Neuron (In)
Alzheimer's disease	Oligo, Neuron (Ex), Neuron (In)
Parkinson's disease	Oligo, Neuron (Ex), Neuron (In)
Huntington's disease	Oligo, Neuron (Ex), Neuron (In)
Phagosome	Mic, Astro
HIF-1 signaling pathway	Mic, Endo
PI3K-Akt signaling pathway	Mic, Endo
Antigen processing and presentation	Mic, Endo
Mineral absorption	Astro, Endo
cAMP signaling pathway	Oligo, Neuron (In)
Protein processing in ER	Astro, Oligo
Gap junction	Astro, Neuron (Ex)
Rap1 signaling pathway	Neuron (In), Endo

Mic: Microglia; Astro: Astrocytes; Oligo: Oligodendrocytes; Neuron (Ex): Excitatory neurons; Neuron (In): Inhibitory neurons; Endo: Endothelial cells

### Distinct molecule and pathways changes in AD

Except for the DEGs that cross different cell types, we also identified the cell type-specific common genes and hub genes in 3 studies and explored their related biological processes and pathways that involve in AD-pathology. Besides the immunometabolism and oxidative stress-related genes, the ribosomal genes, reduced polyubiquitin-related genes-UBC and RPS27A, and genetic risk genes-APOE and APOC1 have also been seen in microglia. The most possible impaired pathways in microglia were the ribosome and PI3K-Akt signaling pathways. Ribosome dysfunction has been regarded as an early event of AD, which may be caused by oxidative damage [101]. Reduced PI3K-Akt signaling pathway has been reported in postmortem AD brain and is closely related to microglia inflammation [102, 103]. These pathways further indicate the microglial oxidation and inflammation in AD. Surprisingly, the endothelial cells were enriched for genes related to immune response and protein ubiquitination and were also involved in antigen processing and presentation. It has been seen the amyloid deposition around cerebral vessels in the preclinical stage of AD, which could impair the BBB integrity and further lead to T cell infiltration and activation in the brain [25, 28, 50, 104, 105]. Thus, the microglia and endothelial cells are the main roles that contributed to the inflammation in AD brain.

While in astrocytes, the most significant transcriptional changes were associated with neuronal signal transduction and extracellular matrix organization. The main impaired pathways were glutamatergic and GABAergic synapses, cell adhesion molecules, ECM-receptor interaction, and mineral absorption in astrocytes. Extracellular matrix promotes the formation of neural networks [106]. Therefore, the astrocytes lost the function to support neuronal activity and maintain brain homeostasis in AD, which has been seen in oligodendrocytes as well [27, 28, 107]. Moreover, the estrogen signaling pathway was impaired in both astrocytes and oligodendrocytes. Reduced expression of estrogen receptors has been reported in hippocampal neurons of AD patients [108]. Restore the estrogen-related signaling could be an effective therapy for the treatment of AD [99]. To be noticed, the transcriptomic alterations in oligodendrocytes, excitatory neurons, and inhibitory neurons could be not specific to AD, but partially overlap with gene sets related to neurodegenerative diseases, such as Parkinson’s disease (PD) and Huntington’s disease (HD). Aberrant myelination and alteration in oligodendrocytes have been identified as common pathophysiological features of AD, PD, and HD [109–112]. Additionally, it’s well known that progressive deficit of structure or dysfunction of neurons and neuronal loss are the main characteristics of neurodegeneration.

We also identified the dysregulated pathways in specific cell types across studies, for instance, ribosome and PI3K-Akt signaling pathway in microglia, cell adhesion molecules, glutamatergic synapse, ECM-receptor interaction, and estrogen signaling pathway in astrocytes, estrogen signaling pathway in oligodendrocytes, endocytosis in excitatory neurons, oxidative phosphorylation in inhibitory neurons, and antigen processing and presentation in endothelial cells.

## Conclusion

In summary, except for the consensus alteration of mitochondrial genes, there are no DEGs that can cross all cell types. Our comprehensive study provides the precise cellular changes, points out the complexity of the transcriptional network in AD pathology, and further highlights the value of cell type-specific transcriptomic analysis. The identified pathogenic genes and functional pathways in AD brain may provide a helpful resource for future investigations and serve as therapeutic targets and biomarkers for the disease.

## Supplementary Information

**Additional file 1: Supplementary File 1.** DEGs are identified and used in microglial cells. **Supplementary File 2.** DEGs are identified and used in astrocytes. **Supplementary File 3.** DEGs are identified and used in oligodendrocytes. **Supplementary File 4.** DEGs are identified and used in excitatory neurons. **Supplementary File 5.** DEGs are identified and used in inhibitory neurons. **Supplementary File 6.** DEGs are identified and used in endothelial cells.

## Data Availability

The snRNA-seq data were obtained from the Supplementary files of 2 articles (Mathys et al., [29]; Lau et al., [28]) and single-cell atlas (http://adsn.ddnetbio.com). All data generated or analysed during this study are included in this published article.
