# Supplementary material for: Cell type-specific potential pathogenic genes and functional pathways in Alzheimer’s Disease
Source: BMC Neurol. 2021 Oct 2;21:381. doi: 10.1186/s12883-021-02407-1 (PMC8487122; doi:10.1186/s12883-021-02407-1)
Supplement: Supplementary file 1 — Additional file 1: Supplementary File 1. DEGs are identified and used in microglial cells. Supplementary File 2. DEGs are identified and used in astrocytes. Supplementary File 3. DEGs are identified and used in oligodendrocytes. Supplementary File 4. DEGs are identified and used in excitatory neurons. Supplementary File 5. DEGs are identified and used in inhibitory neurons. Supplementary File 6. DEGs are identified and used in endothelial cells. [file 12883_2021_2407_MOESM1_ESM.xlsx]

**Supplemental Information**

**Cell type-specific potential pathogenic genes and functional pathways in Alzheimer’s Disease**

Xiao-Lan Wang^1,*^, Lianjian Li^2,*^

^1^Université de Strasbourg, Laboratoire de Neuroscience Cognitives et Adaptatives (LNCA), Strasbourg, France

^2^Department of Surgery, Hubei Provincial Hospital of Traditional Chinese Medicine, Hubei Province Academy of Traditional Chinese Medicine, Wuhan, China

**Correspondence**

Xiao-Lan Wang M.D. Ph.D.

Laboratoire de Neuroscience Cognitives et Adaptatives (LNCA),

Université de Strasbourg.

12 rue Goethe 67000 Strasbourg, France
Tel: +33 0753474547

E-mail: [xiao-lan.wang@etu.unistra.fr](mailto:xiao-lan.wang@etu.unistra.fr)

ORCID ID: 0000-0002-3549-3680

Supplemental Files

Supplementary File 1. DEGs are identified and used in microglial cells.

Supplementary File 2. DEGs are identified and used in astrocytes.

Supplementary File 3. DEGs are identified and used in oligodendrocytes.

Supplementary File 4. DEGs are identified and used in excitatory neurons.

Supplementary File 5. DEGs are identified and used in inhibitory neurons.

Supplementary File 6. DEGs are identified and used in endothelial cells.
